# Absorbable Plate-Assisted Columellar Strut Graft Using Auricular Cartilage: A Large-Scale Clinical Study of 1710 Cases in Rhinoplasty

**DOI:** 10.3390/jcm15156103

**Published:** 2026-08-05

**Authors:** Hyo Heon Kim, Jae Hoon Kim

**Affiliations:** Department of Plastic and Reconstructive Surgery, Gachon University Gil Medical Center, Incheon 21565, Republic of Korea; jaaaaar888@gilhospital.com

**Keywords:** rhinoplasty, columellar strut graft, absorbable plate, auricular cartilage, nasal tip projection, East Asian

## Abstract

**Background**: Columellar strut grafting is a widely adopted technique for nasal tip support in rhinoplasty; however, autologous cartilage grafts are associated with well-recognized limitations, including resorption, warping, and donor-site morbidity. Absorbable plates composed of poly-L-lactic acid (PLA) or poly-lactic-co-glycolic acid (PLGA), which have an established role in craniofacial fracture fixation, were investigated as a structural adjunct to auricular cartilage columellar strut grafts. The present study evaluates the safety and clinical outcomes of this technique in a large patient cohort. **Methods**: A retrospective review was conducted of 1710 consecutive patients who underwent primary rhinoplasty with absorbable plate-assisted columellar strut grafting between November 2012 and June 2022. A PLA plate (n = 792) or PLGA plate (n = 918) was used in combination with auricular conchal cartilage. Nasal tip projection was assessed using standardized clinical photographs and Ricketts’ E-line analysis at a minimum follow-up of 1 month (range, 1 month to 6 years). **Results**: Satisfactory nasal tip projection was achieved and sustained throughout the follow-up period in the majority of patients. Transient mild overprojection, attributable to intentional overcorrection, resolved consistently by 6 months postoperatively. Among patients with recorded follow-up, the overall complication rate was 0.35% (6 of 1710 patients): 1 case of minor skin exposure and 1 case requiring revision for the aesthetic desire of further tip elevation (rather than structural projection loss) in the PLA group and 4 cases of mucosal penetration in the PLGA group, all of which occurred during the initial learning phase and resolved without sequelae. No delayed infections were recorded. **Conclusions**: Absorbable plate-assisted columellar strut grafting using auricular conchal cartilage is a safe, reproducible, and effective technique for achieving stable nasal tip projection. By providing temporary structural support during the critical early healing phase and subsequently undergoing biologic resorption, this approach mitigates the long-term risks associated with permanent implants while avoiding the morbidity of costal cartilage harvest. Despite the limitations of a retrospective design lacking a cartilage-only control group and objective three-dimensional volumetric assessments, it represents a viable and practical alternative for nasal tip support, particularly in East Asian patients with thick skin envelopes and weak cartilaginous frameworks.

## 1. Introduction

Achieving stable and aesthetically pleasing nasal tip projection remains among the most technically demanding objectives in rhinoplasty. Numerous techniques have been described to provide structural support to the nasal tip, including septal extension grafts, columellar strut grafts, and their various modifications [1,2]. Autologous cartilage—including septal, auricular, and costal cartilage—remains the preferred graft material for nasal tip support owing to its biocompatibility, structural adaptability, and long-term integration [3].

Nonetheless, each autologous cartilage source carries inherent limitations. Historically, in our practice, auricular cartilage was primarily utilized for primary rhinoplasty, while autologous costal cartilage was reserved for revision cases. However, we frequently encountered postoperative challenges with auricular cartilage alone; its inherent lack of rigidity often resulted in a progressive loss of nasal tip projection over time, leading to increased patient dissatisfaction and complaints [4]. Conversely, costal cartilage affords greater structural rigidity but entails significant donor-site morbidity, including the risks of pneumothorax, chest wall scarring, and prolonged postoperative pain, rendering it less suitable for routine primary rhinoplasty [5]. Furthermore, excessive rigidity inherent to certain graft constructs may impair the natural dynamics and tactile compliance of the nasal tip [6].

To circumvent autologous donor-site morbidity, allograft costal cartilage, including irradiated homologous costal cartilage and fresh-frozen cadaveric rib cartilage, has also been used as a structural graft source in primary and revision rhinoplasty [7,8]. These materials may reduce donor-site morbidity compared with autologous costal cartilage, but their use is accompanied by concerns regarding availability, cost, unpredictable resorption, warping, infection, and the quality of long-term evidence.

These challenges are especially pronounced in East Asian patients, who characteristically present with thicker, sebaceous skin envelopes and inherently weaker lower lateral cartilages [9,10]. In this population, simultaneously achieving sufficient tip projection and maintaining long-term structural stability represents a particularly difficult surgical challenge.

In rhinoplasty, the application of absorbable plates demands meticulous patient selection and the establishment of strict surgical indications. Historically, this material has been contraindicated for patients desiring excessive nasal projection, those with weak baseline structural support, or individuals presenting with a thick soft-tissue envelope, particularly at the nasal tip. Recently, the field has witnessed a significant decline in the use of absorbable plates, driven largely by a paradigm shift toward irradiated homologous costal cartilage (IHCC) and the subsequent cessation of plate manufacturing. Despite these unfavorable industry trends, the authors has developed specific technical refinements and a standardized protocol that have yielded highly stable and successful outcomes in a large cohort of patients. This study aims to present these optimized techniques and demonstrate the clinical viability of absorbable plates when rigorous patient selection and specialized surgical know-how are applied.

Absorbable fixation plates—specifically those composed of poly-L-lactic acid (PLA) and poly-lactic-co-glycolic acid (PLGA)—have been employed extensively in craniofacial and orthognathic surgery for rigid fixation of osseous fractures. Resorbable plates have also been described in reconstructive septal surgery, particularly polydioxanone (PDS) foil or plate systems used to stabilize septal cartilage fragments in septoplasty and extracorporeal septal reconstruction [11,12,13]. However, in those applications, the plate primarily functions as a temporary internal splint. The columellar strut graft presents a distinct biomechanical challenge, requiring a load-bearing construct to actively resist continuous downward tip forces.

Absorbable plates provide temporary mechanical support during the healing phase and subsequently undergo predictable hydrolytic degradation, allowing for replacement by endogenous fibrous or cartilaginous tissue. Based on these favorable biologic properties, we postulated that these biomaterials could serve as a temporary structural scaffold in rhinoplasty. By providing adequate tip support during the critical early postoperative period, they effectively circumvent the long-term complications inherent to permanent alloplastic implants. What initially began as an exploratory alternative for patients reluctant to undergo costal cartilage harvesting quickly proved to be highly effective. Ultimately, this evolved into our primary surgical option: a hybrid technique utilizing absorbable plates to reinforce auricular cartilage, simultaneously resolving the structural shortcomings of auricular cartilage and the donor-site morbidities associated with costal grafts.

While offering clear clinical advantages, the application of absorbable plates demands meticulous patient selection and the establishment of strict surgical indications. Historically, this material has been contraindicated for patients desiring excessive nasal projection, those with weak baseline structural support, or individuals presenting with a thick soft-tissue envelope, particularly at the nasal tip. Recently, a paradigm shift toward the increased utilization of irradiated homologous costal cartilage (IHCC) has occurred within the field [14,15]; concurrently, the application of absorbable plates has significantly declined, compounded by the cessation of their manufacturing.

Notwithstanding these unfavorable industry trends and historical limitations, the authors has achieved highly stable and successful outcomes in a large cohort of patients. This study aims to share the author’s clinical experience and demonstrate the sustained viability of absorbable plates when rigorous patient selection and specialized surgical know-how are maintained.

## 2. Materials and Methods

### 2.1. Study Design and Patients

This retrospective study was conducted in accordance with the ethical standards of the Declaration of Helsinki and was approved by the Institutional Review Board of Gachon University Gil Medical Center (IRB No. GDIRB2026-176). Given the retrospective design of the study and the use of a completely de-identified surgical database, the IRB granted a waiver of the requirement for written informed consent for research purposes. As the surgeries were performed by a single surgeon across multiple clinical institutions over the study period, written authorization for the retrospective use of patient records was obtained from the heads of the currently active participating institutions and submitted to the Gachon University Gil Medical Center IRB. For cases performed at an international institution that has since ceased operations, rendering administrative authorization unobtainable, the utilized data were strictly limited to completely de-identified numerical aggregate counts. The entire data collection protocol, including the use of data from the closed institution, was formally reviewed and approved by the aforementioned IRB. It should be noted that while research consent was waived, standard written informed consent for the surgical procedure itself had been obtained from all patients prior to surgery at each respective site. The study population comprised 1710 consecutive patients who underwent primary open rhinoplasty with absorbable plate-assisted columellar strut grafting, performed by a single surgeon using an identical standardized technique across these multiple clinical settings, between November 2012 and June 2022. This study cohort (n = 1710) includes every single consecutive case, representing a 100% share of the surgeon’s combined primary rhinoplasty volume across these institutions during this decade-long period, as this was his exclusive, standardized technique for primary rhinoplasty.

In this retrospective study of 1710 consecutive patients, structural safety and complication rates were assessed for the entire cohort based on direct clinical examinations and medical records. For the objective morphological assessment of nasal tip projection using Ricketts’ E-line analysis on standardized photographs, the cohort was strictly limited to 820 patients (792 in the PLA group and 28 in the PLGA group) treated at currently active domestic institutions where retrospective photographic data access was officially authorized by the IRB. The remaining 890 patients (all in the PLGA group) treated at an international institution were evaluated solely based on de-identified aggregate clinical data, as administrative closure of the facility precluded the export of photographic records.

The minimum follow-up period was 1 month (range, 1 month to 6 years). Furthermore, the entire study population consisted of East Asian individuals, who characteristically present with thicker and more sebaceous nasal soft tissue envelopes compared to other ethnicities. Therefore, baseline skin type was considered uniformly thick across the cohort.

### 2.2. Materials

**Figure 1:**
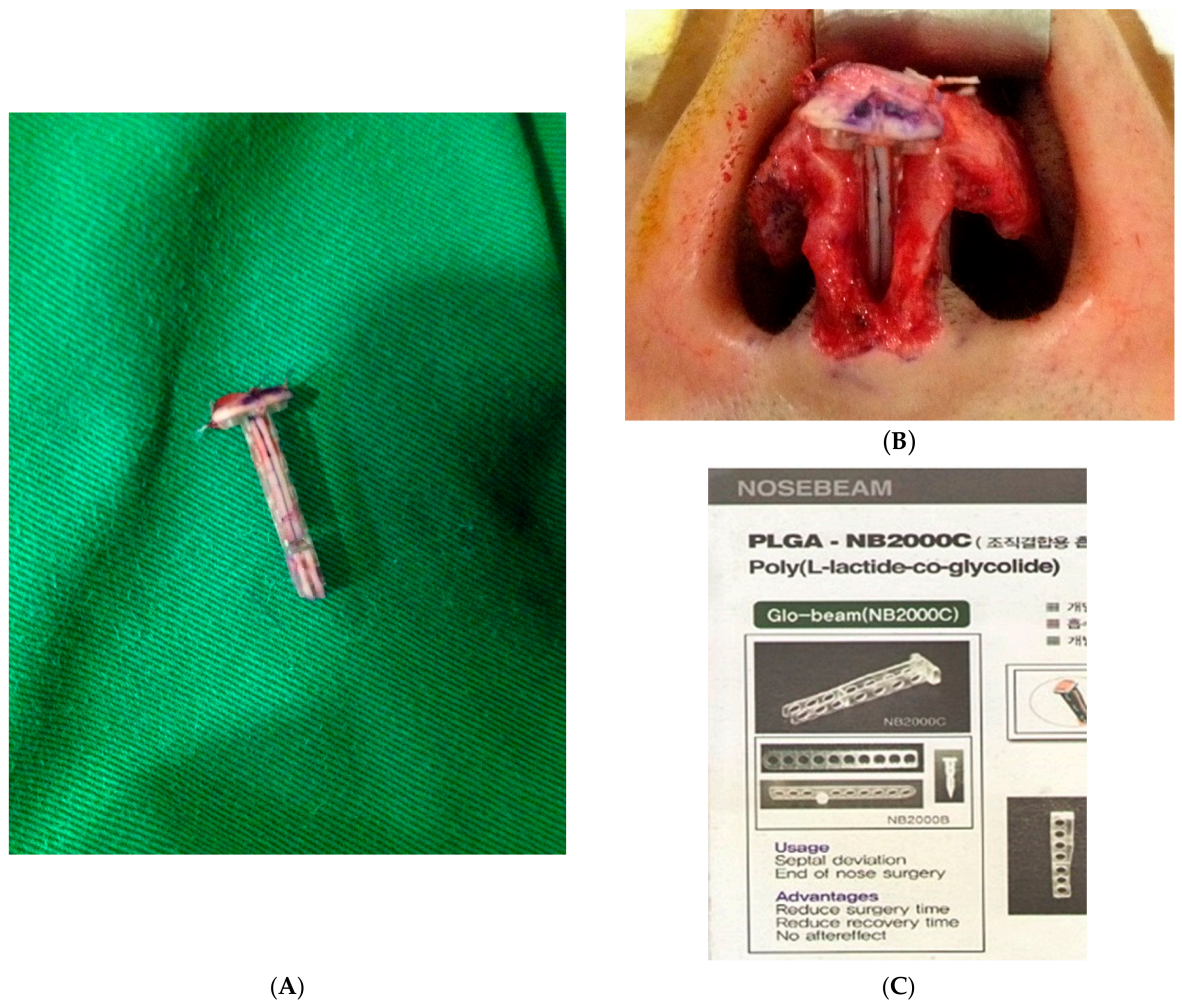
Illustration of the PLA plate (Nosebeam) with pre-designed groove for auricular cartilage insertion (**A**) and the assembled columellar strut complex prior to implantation (**B**). The PLA plate provides structural support as an alloplastic scaffold, while the auricular cartilage carved to fit the groove serves as a biologic reinforcement, together forming the columellar strut complex. (**C**) An archival photograph of the absorbable plate, provided for reference as the product is no longer commercially manufactured. (Source: http://www.maymedi.com/goods/goods_view.php?goodsNo=1000000993 accessed on 15 July 2026).

**Figure 2:**
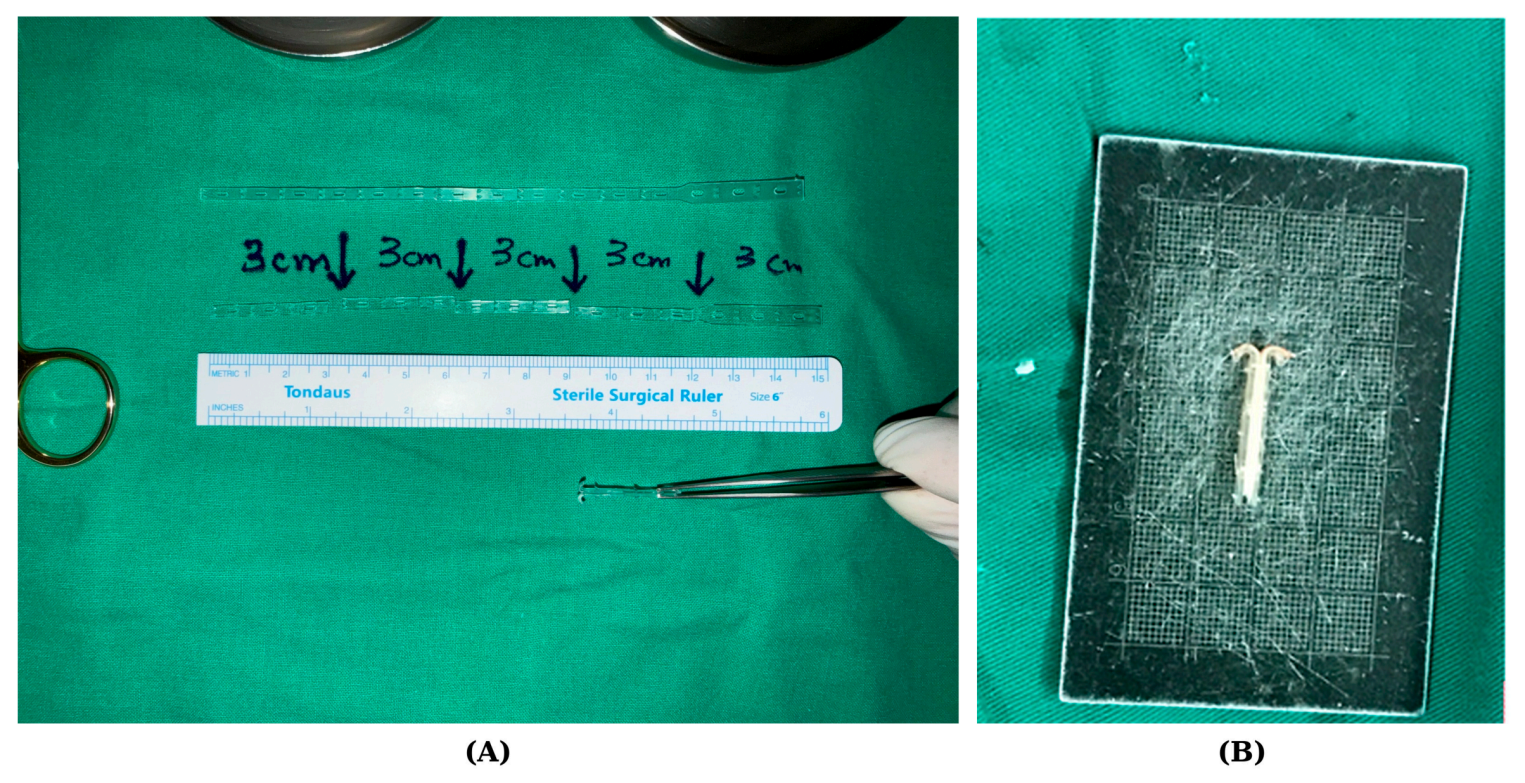
Photographs of the PLGA absorbable plate (Weilamei) and the assembled composite columellar strut construct. (**A**) The Weilamei PLGA plate after trimming into representative operative lengths, shown with a surgical ruler for scale. The plate was trimmed to approximately 3 cm according to the desired columellar height and nasal tip projection, and the fenestrated strip design allowed further intraoperative adjustment and suture fixation. (**B**) The completed absorbable plate–auricular cartilage complex assembled on a measurement grid. The harvested conchal cartilage was apposed to the thermally molded PLGA plate and secured with absorbable sutures, forming the composite columellar strut construct ready for insertion between the medial crura. (Weilamei; Zhejiang Wedu Medical Co., Ltd., Dongyang, China).

Two commercially available absorbable plate systems were utilized in this series:

(1) A PLA plate (Nosebeam^®^; Seoul, Republic of Korea) was used in 792 patients. This device features a pre-formed longitudinal groove designed to facilitate secure insertion and stable fixation of the auricular cartilage graft. As the material is no longer commercially manufactured, an archival photograph is provided in Figure 1.
(2) A PLGA plate (Weilamei^®^; Zhejiang, China) was used in 918 patients. Both devices received regulatory approval from their respective national authorities prior to clinical use. (Figure 2) The PLGA plate dimensions before trimming were: length 30 mm × width 3 mm × thickness 1 mm, with 4 perforations.

### 2.3. Surgical Technique

Auricular cartilage was harvested from the conchal bowl via a standard anterior approach. Meticulous attention was paid to preservation of the antihelix, concha cymba, and conchal rim to prevent donor-site contour irregularity or deformity.

For the PLA plate, the harvested auricular cartilage was inserted into the pre-formed groove of the plate, creating a composite columellar strut construct. The exact original dimensions of the Nosebeam^®^ plate can no longer be specified, as the product is no longer commercially manufactured and archival technical specifications are unavailable. For the PLGA plate, the material was trimmed to an appropriate length of approximately 3 cm and thermally remolded following brief immersion in sterile physiologic saline heated to a temperature exceeding 90 °C, which renders the plate pliable for contouring.

Regardless of the specific material used (PLA or PLGA), the absorbable plate was first immersed in sterile physiologic saline heated to a temperature exceeding 90 °C. This brief thermal exposure renders the plate highly pliable, allowing it to be custom-molded to match the specific anatomic requirements and aesthetic goals of the patient. The length of the plate was meticulously calibrated based on the patient’s desired nasal tip projection, incorporating a mandatory intraoperative overcorrection ranging from 2 to 5 mm. The precise magnitude of this overcorrection was determined by a dynamic intraoperative assessment of structural integrity, specifically evaluating the inherent stiffness of the medial crura of the lower lateral cartilages (which are sutured bilaterally to the plate) and the rigidity of the harvested auricular cartilage. In cases where both the medial crura and the auricular cartilage provided robust support, a conservative overcorrection of 2 mm was deemed sufficient. Conversely, when both structures exhibited weak supportive capacity, a maximal overcorrection of 5 mm was applied. This deliberate 5 mm overprojection anticipates the progressive partial resorption of the plate and the subsequent settling of the soft tissue envelope, ensuring the nasal tip descends precisely to the patient’s targeted height by the 6-month postoperative mark.

After cooling to body temperature, the molded plate was apposed to the auricular cartilage graft and secured with 4−0—absorbable sutures to form the final composite construct. To prevent the natural tendency of auricular cartilage to curl or warp, the cartilage was meticulously carved, thinned, and perfectly adapted to the plate. It was then firmly sutured flat against the rigid absorbable plate, effectively neutralizing its natural curl and maintaining a stable, simple linear strut.

The composite graft was introduced into the columellar space and positioned between the medial crura under direct vision through an open rhinoplasty approach. Fixation was achieved with transcolumellar absorbable mattress sutures. In cases requiring significant tip elevation (e.g., maximal overcorrection), additional structural modifications were mandated to prevent specific complications. First, to mitigate the increased tension on the skin envelope, a widened auricular cartilage cap graft was utilized, and a layer of artificial dermis or autologous dermis was routinely interposed over the cap graft for soft tissue protection. Second, because significant anterior projection inherently induces cephalic rotation (potentially resulting in a short, upturned nose deformity), a derotation graft using auricular cartilage was meticulously and rigidly sutured in place to establish secure caudal vector control.

According to our institutional protocol for this procedure, pre-operative and intra-operative prophylactic antibiotics were not routinely administered. Instead, patients received a targeted post-operative regimen comprising a second-generation cephalosporin and a fluoroquinolone (ciprofloxacin). Depending on the individual clinical presentation, patients were treated with **either** intravenous (IV) antibiotics for up to 3 days **or** an oral regimen of the same antibiotics for 5 days.

To reduce mucosal penetration during the early learning phase, the inferior and superior edges of the plate were rounded, sharp corners were avoided, and the construct was seated without excessive caudal pressure against the columellar skin or intranasal mucosa. After these technical refinements, no further cases of skin exposure or mucosal penetration were observed.

### 2.4. Outcome Evaluation

**Figure 3:**
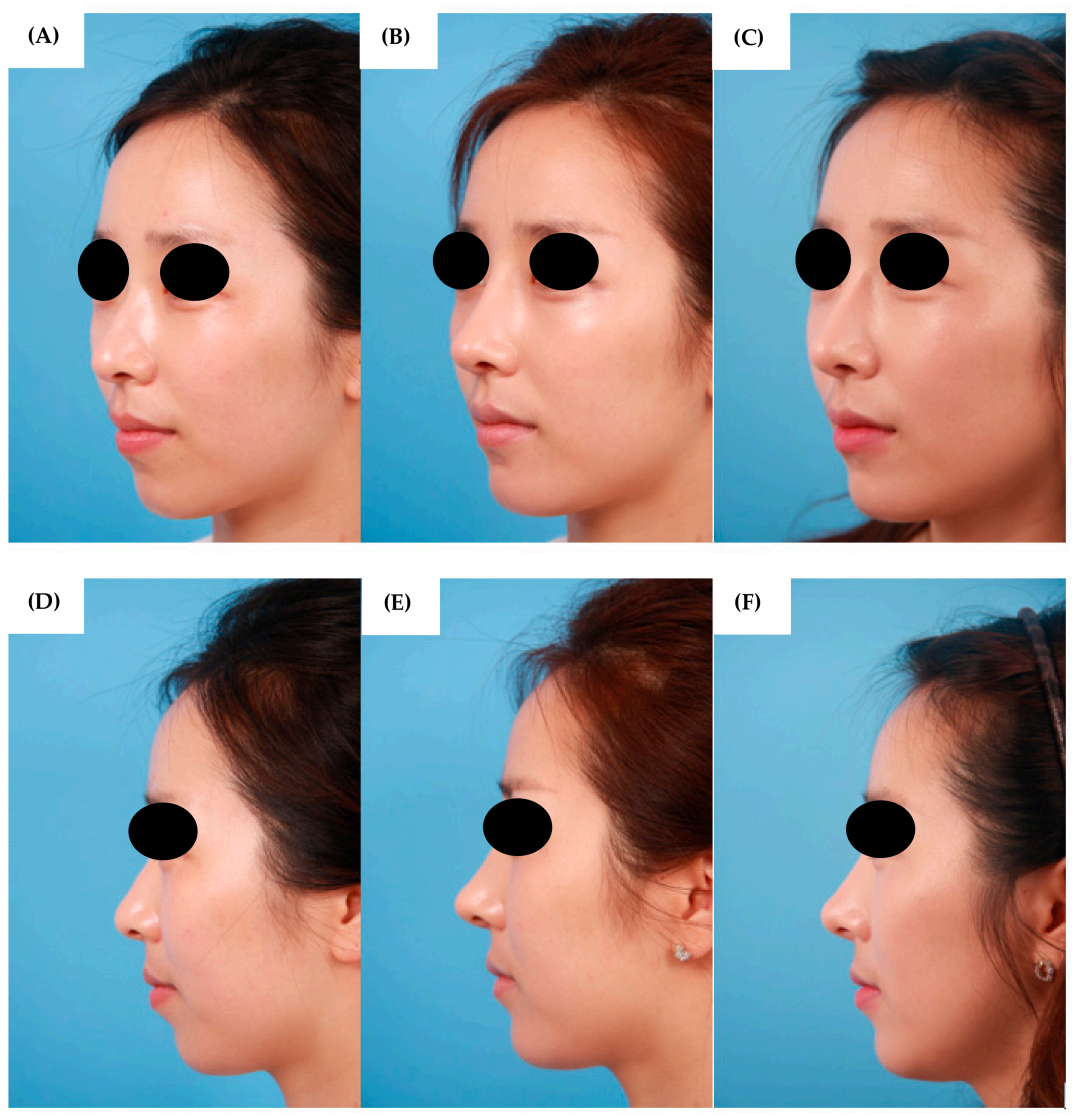
Representative preoperative and postoperative clinical photographs of a patient in the PLGA group. Oblique views obtained preoperatively (**A**), at 1 month (**B**), and at 11 months (**C**) following absorbable plate–assisted columellar strut grafting. Corresponding lateral views at the same time points are shown in (**D**), (**E**), and (**F**), respectively. Transient mild overprojection observed in the early postoperative period resolves progressively, with stable and natural nasal tip contour achieved by 11 months postoperatively.

**Figure 4:**
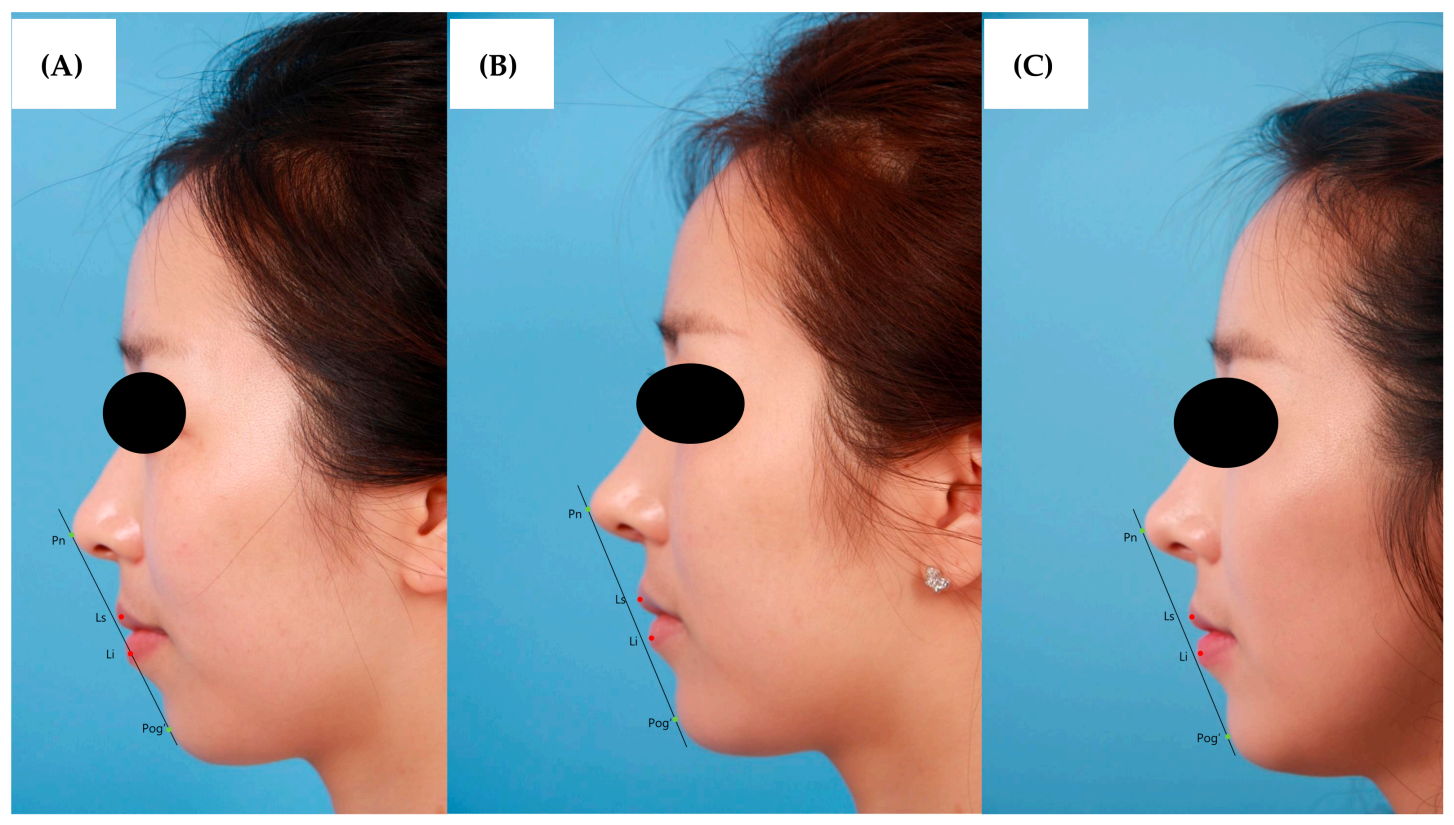
Serial lateral photographs with Ricketts’ E-line analysis demonstrating the postoperative course following absorbable plate–assisted columellar strut grafting. Measurements were obtained preoperatively (**A**), at 1 month (**B**), and at 11 months (**C**). Preoperatively (**A**), the nasal tip (Pn) was underprojected relative to the E-line. Reflecting the intentional overcorrection of 2 to 3 mm performed intraoperatively, mild residual overprojection persisted at 1 month (**B**), with Pn positioned approximately 1 to 2 mm anterior to the E-line. By 11 months, the nasal tip projection had normalized, with Pn positioned at or within 1 mm of the E-line, consistent with the aesthetic ideal for East Asian patients. The upper lip (Ls pink dot) and lower lip (Li pink dot) maintained stable relationships to the E-line throughout the follow-up period.

Clinical outcomes were assessed using standardized photographs obtained in frontal, lateral, and basal views at defined postoperative intervals (1 week, 1 month, 3 months, 6 months, 1 year, and annually thereafter) (Figure 3). Nasal tip projection was quantitatively evaluated using Ricketts’ E-line analysis on standardized lateral photographs (Figure 4). All complications—including surgical site infection, implant or graft exposure, and mucosal penetration—were recorded. The requirement for unplanned revision surgery was also systematically documented. Three-dimensional face scanning and validated patient-reported outcome measures (PROMs) were not routinely available during the early study period; therefore, objective assessment relied on standardized photographic analysis. This limitation is acknowledged in the Discussion. 

## 3. Results

A total of 1710 patients were included in the analysis: 792 in the PLA group and 918 in the PLGA group. Baseline demographic characteristics and clinical outcome data for both groups are summarized in Table 1.

**Figure 5:**
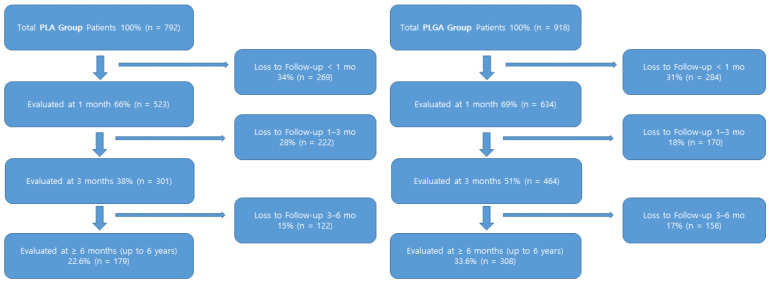
Flow diagrams illustrating patient retention and attrition rates over the postoperative follow-up period. The early PLA group cohort (n = 792) demonstrates a substantial early loss to follow-up, typical of satisfied patients in aesthetic rhinoplasty practice. The later PLGA group cohort (n = 918) shows a notably improved retention rate at the evaluation of 6-month and beyond evaluation milestone (33.6% compared to the PLA group), reflecting the successful institutional implementation of a more stringent longitudinal follow-up protocol.

Detailed patient retention and attrition (loss to follow-up) flows across different postoperative intervals for both cohorts are illustrated in Figure 5. As expected in private aesthetic practice, a substantial portion of patients who achieved early satisfaction did not return for long-term evaluations. However, following the institutional implementation of stricter follow-up protocols, the retention rate for patients strictly reaching the 6-month or a longer evaluation milestone significantly improved in the later PLGA compared to the early PLA.

Satisfactory nasal tip projection was achieved in the vast majority of patients and was maintained throughout the follow-up period. Transient mild overprojection was consistently observed during the early postoperative phase, in accordance with the planned intraoperative overcorrection strategy of 2 to 5 mm. This normalized progressively, with patients demonstrating a natural and aesthetically appropriate nasal tip contour by approximately 6 months postoperatively.

**Figure 6:**
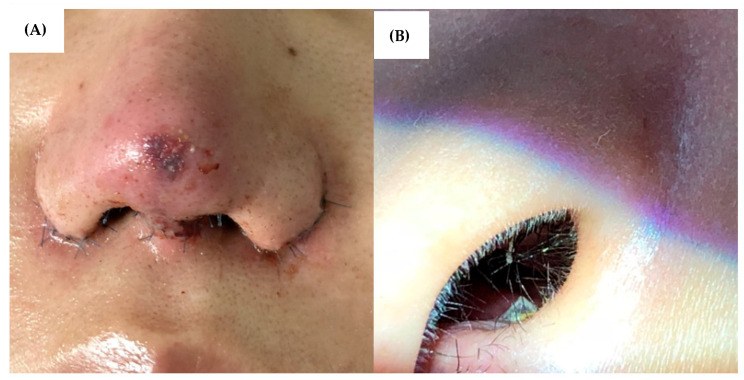
Representative photographs of complications encountered during the learning phase. (**A**) Minor columellar skin exposure observed in the PLA group, which resolved without surgical intervention. (**B**) Intranasal mucosal penetration in the PLGA group, managed conservatively without sequelae.

The overall complication rate was 0.35%, with 6 adverse events identified among 1710 patients. In the PLA group, 1 case of minor columellar skin exposure and 1 case requiring revision due to the patient’s aesthetic desire for further tip elevation—rather than structural projection loss—were recorded (2 of 792; 0.25%). In the PLGA group, 4 cases of intranasal mucosal penetration were identified (4 of 918; 0.44%) (Figure 6). All complications were encountered during the initial learning phase of the procedure and were managed successfully without sequelae. Notably, no complications of this nature were observed following the aforementioned technical refinement of the operative technique (e.g., rounding the plate edges). No cases of delayed infection were identified at any point during the follow-up period.

During the single operating surgeon’s 5-year tenure at the international institution, a total of 890 patients underwent this procedure within that specific sub-cohort, a volume naturally driven by positive patient feedback and a consistent safety record. Routine clinical follow-ups over this extended period confirmed sustained structural stability, with no observed cases of structural failure, loss of projection, or delayed infection.

**Figure 7:**
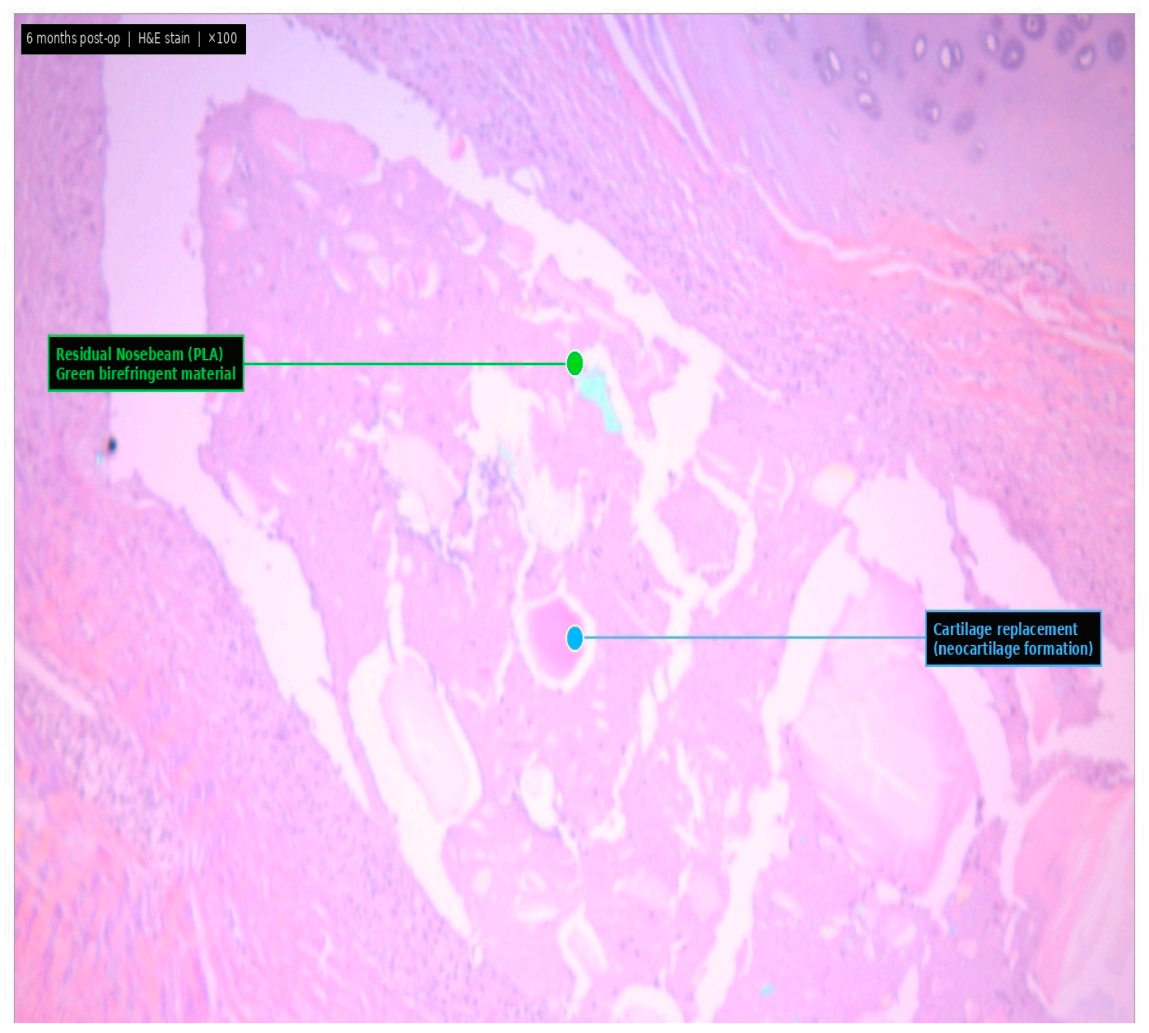
Histological examination (H&E stain, ×100) of the columellar strut complex retrieved 6 months postoperatively from a patient who underwent revision surgery for further tip elevation. The green birefringent material represents residual undegraded Nosebeam (PLA plate), confirming its slow biodegradation profile at 6 months. The surrounding tissue demonstrates active replacement by nascent cartilage, indicating progressive chondrogenesis around the PLA scaffold. This revision was an elective secondary aesthetic augmentation for additional tip elevation and was not classified as failure of projection maintenance or revision for inadequate projection loss.

Importantly, no patient required secondary surgery for structural failure or inadequate tip projection, and no case necessitated conversion to costal cartilage grafting. Regarding the aforementioned revision case in the PLA group, the patient underwent elective revision for further tip elevation despite having maintained projection relative to the preoperative goal. This case was not categorized as revision for inadequate projection loss because the original postoperative projection target had been achieved, and the revision was performed for additional aesthetic augmentation at the patient/surgeon request. The histologic specimen from this elective revision is shown in Figure 7.

Comparative clinical observation between the two plate types indicated that PLGA plates maintained structural support over a more prolonged postoperative period than PLA plates, a finding consistent with the known differences in their respective in vivo degradation kinetics.

## 4. Discussion

Autologous cartilage grafting remains the gold standard for structural nasal tip support; however, its limitations are well established in the literature. Progressive resorption, warping, and inadequate long-term projection are recognized challenges with both septal and auricular cartilage, and are particularly consequential in East Asian patients who present with thick, sebaceous skin envelopes and structurally deficient lower lateral cartilages [8,9,16,17]. The present study demonstrates that absorbable plate-augmented columellar strut constructs can reliably provide the structural support necessary during the critical early healing phase, after which their gradual hydrolytic degradation permits progressive biologic remodeling and replacement by endogenous fibrous or cartilaginous tissue.

Permanent alloplastic implants, such as expanded polytetrafluoroethylene and porous high-density polyethylene, have been employed as alternatives to autologous grafts for nasal augmentation; however, their use is associated with well-documented long-term risks, including implant infection, extrusion, capsular contracture, and chronic foreign body reaction [18]. Absorbable materials inherently circumvent these risks through complete biologic resorption, thereby eliminating the permanent foreign body burden. The complication rate of 0.35% observed among patients with recorded follow-up in the present series is consistent with a favorable biocompatibility and soft-tissue tolerance profile for PLA- and PLGA-based plates. However, given the inherent loss to follow-up in this retrospective aesthetic cohort, this figure should not be interpreted as a population-level incidence estimate (see Limitations).

The reproducible postoperative course observed in this series represents a clinically important advantage of the technique. Transient mild overprojection in the immediate postoperative period consistently resolved to a natural contour by approximately 6 months, validating the rationale for systematic intraoperative overcorrection by 2 to 5 mm depending on the structural integrity of the medial crura and auricular cartilage. This predictable trajectory was observed consistently across both plate types and throughout the study period, enabling standardized preoperative counseling and reliable surgical planning. It is important to note that this procedure is limited strictly to female patients, as the pronounced nasal tip projection it creates is typically unacceptable to the male aesthetic preference. While two anatomically male patients in the PLGA cohort did undergo this surgery, both were transgender individuals who identified with female gender characteristics.

The differential degradation kinetics of PLA and PLGA plates carry meaningful clinical implications for material selection. PLGA undergoes hydrolytic degradation via simultaneous bulk erosion and surface hydrolysis, a biphasic process that results in a longer duration of mechanical integrity compared with PLA alone [14]. This distinction in resorption profile may guide individualized material selection based on the anticipated magnitude of tip projection required, skin envelope thickness, and surgeon preference, particularly in patients with greater structural demands.

The existing literature on resorbable materials in nasal surgery has been largely confined to septal reconstruction. Boenisch and Mink demonstrated the utility of polydioxanone (PDS) foil in external septoplasty, reporting successful stabilization of septal cartilage fragments with complete resorption by 25 weeks and no adverse tissue reaction [10]. Subsequent studies further confirmed that resorbable foils promote cartilage regeneration without inducing secondary deviation, and larger clinical series have reported their successful application in extracorporeal septoplasty for the correction of complex septal deformities [11,12]. However, a critical distinction exists between septal reconstruction and columellar strut grafting. In septal surgery, the resorbable material functions essentially as a passive fixation scaffold—it holds pre-existing cartilage fragments in a corrected position against a largely self-supporting nasal framework, reinforced on multiple sides by the surrounding mucosal envelope and adjacent bony structures. The mechanical demands in this context are comparatively modest.

In contrast, the columellar strut graft must actively resist the continuous downward and posterior forces exerted by the nasal tip soft tissue envelope—forces that are substantially greater in East Asian patients with characteristically thick, sebaceous skin and structurally deficient lower lateral cartilages. In this biomechanical context, the resorbable plate serves not merely as a fixation aid, but as a true structural load-bearing element that must maintain adequate mechanical integrity throughout the critical early healing phase. To the best of our knowledge, no prior study has evaluated the use of resorbable plates specifically in this load-bearing columellar application, nor has any series reported outcomes across a patient cohort of comparable size. The present study, encompassing 1710 consecutive cases, with follow-up data available for a subset of patients extending to 6 years, therefore addresses a clinical scenario that the existing septoplasty literature does not speak to, and provides preliminary evidence, to be confirmed by the planned prospective follow-up study, that PLA- and PLGA-based plates are capable of fulfilling this more demanding structural role without adverse sequelae.

Other tools and grafting strategies described for similar reconstructive goals include cartilage-only columellar struts, septal extension grafts, autologous costal cartilage, allograft costal cartilage, permanent alloplastic implants, and PDS plate/foil-assisted septal reconstruction. Compared with cartilage-only struts, the present construct was designed to provide greater temporary rigidity during early healing. Compared with autologous costal cartilage, it avoids chest donor-site morbidity. Compared with allograft costal cartilage, it avoids reliance on tissue-bank grafts while preserving autologous biologic coverage through auricular cartilage. Compared with permanent alloplastic implants, the absorbable plate reduces long-term permanent foreign-body burden. Compared with PDS plates used in septal reconstruction, PLA/PLGA plates in this study were used in a more load-bearing columellar position rather than as passive septal splints.

Despite the comparative advantages and high clinical stability demonstrated in our series, the authors has discontinued the use of absorbable plates since 2022. This decision was not due to an inherent failure of the material itself, but rather driven by significant extrinsic market and industry shifts. First and foremost, the material is no longer commercially manufactured. In the past, indiscriminate application of these plates in suboptimal candidates by inexperienced practitioners resulted in a high incidence of complications elsewhere. This ultimately led to widespread rejection by mainstream plastic surgeons and the inevitable bankruptcy of the manufacturing company. Simultaneously, the surgical trend shifted decisively toward irradiated homologous costal cartilage (IHCC). The decreased acquisition cost of IHCC, combined with a growing consensus regarding its safety profile and ease of use, accelerated the obsolescence of absorbable plates. Nevertheless, the extensive, large-scale clinical success achieved through the author’s proprietary surgical protocols proves that absorbable plates can be highly reliable when utilized correctly. By documenting and sharing this comprehensive experience, the authors intends to highlight the material’s latent potential. This report serves to preserve these technical refinements within the academic literature, intentionally leaving the door open for the future exploration and evolution of alternative surgical modalities.

Several limitations of this study merit acknowledgment. First, because this was a retrospective study, a concurrent cartilage-only control group was not available; thus, the low complication rate and stable photographic outcomes should be interpreted as supportive observational evidence rather than definitive proof of superiority over conventional cartilage-only columellar strut grafting. This design choice reflects the clinical rationale underlying adoption of the plate-assisted technique: in our prior experience with cartilage-only auricular grafts, a substantial proportion of patients developed progressive loss of nasal tip projection over time, resulting in increased dissatisfaction and revision requests. The primary aim of the present study was therefore to introduce and validate this plate-assisted technique as a safe and reproducible alternative, rather than to conduct a head-to-head comparative trial; a prospective comparative study incorporating a cartilage-only control arm is currently being planned as a subsequent investigation.

Second, objective outcome assessment using Ricketts’ E-line on clinical photographs could not be performed uniformly across the entire cohort. Due to the administrative closure of the international institution, photographic data for 890 patients in the PLGA group could not be utilized. However, the operating surgeon’s 5-year tenure at the international facility allowed for consistent and long-term outpatient follow-up. While the lack of objective photographic data for this subset remains a limitation, this continuous clinical monitoring suggests that the aggregate data regarding the absence of structural complications may still provide meaningful clinical insights into the safety of this sub-cohort. Furthermore, we acknowledge that even for the cases where photographic analysis was possible, the evaluation could have been significantly strengthened by the routine use of objective three-dimensional (3D) facial scanning for volumetric analysis, as well as validated patient-reported outcome measures (PROMs) such as the FACE-Q or Rhinoplasty Outcome Evaluation (ROE) questionnaire.

Third, loss to follow-up is an inherent concern in retrospective aesthetic surgery cohorts. Although the early safety profile is robustly supported by the entire cohort with a minimum 1-month follow-up, the number of evaluable patients who strictly reached the 6-month morphological assessment mark naturally decreased. This attrition should be considered when interpreting the reported structural durability up to 6 years. Consequently, the reported complication rate of 0.35% reflects outcomes among patients who returned for follow-up evaluation and should not be interpreted as a true population-level incidence estimate for the full operative cohort, since patients who experienced complications or dissatisfaction may have sought care elsewhere or otherwise not returned for evaluation.

Fourth, this series was generated within a single-surgeon, high-volume private-practice settings. While the standardized surgical protocol partly explains the internal consistency and low complication rate observed, the homogeneous ethnic composition and single-operator design may limit the generalizability of the findings to multi-surgeon or more ethnically diverse clinical settings. Future prospective, randomized, controlled trials incorporating costal cartilage-based methods, standardized 3D imaging, and validated PROMs across multi-institutional cohorts are warranted to confirm these findings and establish evidence-based indications for this technique.

While our documented complication rate is exceptionally low at 0.35%, this figure must be interpreted with clinical humility. In a private aesthetic practice setting, it is highly probable that some patients who experienced complications or aesthetic dissatisfaction elected to seek revision surgery at other institutions, while others may have failed to return simply due to geographic distance. The cohort with prolonged evaluation likely represents a subset of patients with high baseline satisfaction and established trust in the operating surgeon. Therefore, the primary message of this study is not to claim a near-zero complication rate, but rather to highlight a pragmatic clinical reality: when combined with rigorous patient selection, meticulous technique, and an understanding of its biomechanical properties, the absorbable plate serves as a highly reliable and valuable addition to the plastic surgeon’s armamentarium for nasal tip contouring.

## 5. Conclusions

The findings of the present study demonstrate that absorbable plate-assisted columellar strut grafting using auricular conchal cartilage is a safe, reproducible, and clinically effective technique for achieving stable nasal tip projection in primary rhinoplasty. In this consecutive series of 1710 patients, follow-up data were available for the majority of patients during the early postoperative period, with a subset followed up to 6 years. Among patients with recorded follow-up, the technique yielded a low complication rate of 0.35%, with no cases of delayed infection; as detailed in the Limitations, this rate should be interpreted in light of the inherent loss to follow-up in this retrospective aesthetic cohort. By conferring temporary mechanical support during the early healing phase and permitting subsequent biologic remodeling, this approach offers a rational balance between structural rigidity and long-term adaptability—addressing the principal limitations of both conventional autologous cartilage grafts and permanent alloplastic implants. Importantly, it eliminates the need for costal cartilage harvest, thereby avoiding the associated donor-site morbidity and operative complexity. Although the retrospective cohort design of this study inherently carries certain methodological limitations, the present technique may serve as a valuable and reproducible surgical option for nasal tip support, with particular applicability in East Asian patients requiring reliable, naturalistic tip projection.

## Figures and Tables

**Table 1:** Summary of Patient Demographics and Outcomes by Absorbable Plate Type.

Characteristic	PLA Group (n = 792)	PLGA Group (n = 918)
Study period	November 2012–December 2017	January 2018–June 2022
Follow-up range	1 month–6 years	1 month–6 years
Mean follow-up (months)	1.8 ± 0.8	4.2 ± 0.5
≥6-month Follow-up, n (%)	179 (22.6%)	308 (33.6%)
Total complications, n (%)	2 (0.25%)	4 (0.44%)
Skin exposure	1	0
Mucosal penetration	0	4
Acute & Delayed infections	0	0
Revision for aesthetic tip elevation	1	0
Duration of structural support	Shorter	More prolonged
Age, years, mean ± SD (range)	26.5 ± 4.2 (18–38)	28.8 ± 5.6 (18–45)
Sex, female/male	792/0	916/2

PLA, poly-L-lactic acid; PLGA, poly-lactic-co-glycolic acid. Note: Skin type was not sub-stratified in this table, as the entire cohort consisted exclusively of East Asian patients who characteristically shared a uniformly thick and sebaceous nasal soft tissue envelope. 2 male patients are transgender.

## Data Availability

The data presented in this study are available on request from the corresponding authors. The data are not publicly available due to privacy restrictions.
